# On professional mutualism: a blueprint for early-career virologists

**DOI:** 10.1128/jvi.01156-24

**Published:** 2024-12-16

**Authors:** Priya S. Shah

**Affiliations:** 1 Department of Chemical Engineering, University of California 43348 , Davis, California, USA; 2 Department of Microbiology and Molecular Genetics, University of California 43348 , Davis, California, USA; Indiana University Bloomington, Bloomington, Indiana, USA

**Keywords:** early career, tenure track

## Abstract

Are you an early-career virologist looking for an independent position? Are you searching for the right home for your lab and not sure what you are looking for? I am here to tell you that the right professional home may not be what you expected. The key is to find a home that offers professional mutualism—it allows you and your home department to grow in new directions and hopefully thrive in the process.

## INTRODUCTION

As virologists, we are constantly studying symbiosis. Viruses are obligate intracellular parasites and symbionts as a result. Symbiotic virus-host relationships can be antagonist, neutral, or mutualistic for the host (1). It is these mutualistic relationships that are perhaps the most curious. How can a virus, which uses cellular resources, *benefit* a host? It all depends on context and the right conditions for mutualism to emerge. However, we should not stop at understanding virus-host mutualism from a scientific perspective. We should also consider mutualistic symbiotic relationships in our careers. The right conditions for professional mutualism in virology may surprise you.

I am a professor at the University of California Davis. My research group studies virus-host protein interactions and how these interactions impact virus replication and pathogenesis. We use a combination of quantitative systems biology approaches and classical techniques to dissect these interactions with molecular resolution (2). Many virologists I meet assume that I am a faculty member of a microbiology or cell biology department. In fact, I belong to the departments of Chemical Engineering and Microbiology and Molecular Genetics. The Microbiology and Molecular Genetics department makes sense to most people given the molecular and fundamental nature of my research (it is not clinical, translational, or heavily *in vivo*). However, the Chemical Engineering department raises quite a few eyebrows, especially since it is my primary appointment.

The usual comments and questions follow this revelation:

*But you do virology!* It is true, on the surface, my lab looks mostly like a virology lab. We are funded by the NIH and DOE for fundamental virology research. We publish in virology- and microbiology-focused journals like JVI, mBio, and PLOS Pathogens. But I am a chemical engineer by training. I have not one but *two* degrees in chemical engineering. TL;DR: I have found a great home in Chemical Engineering *and* Microbiology and Molecular Genetics.

*What does chemical engineering have to do with virology?* Chemical engineering and virology have *nothing* in common at the introductory level. However, I found that bringing together two disciplines in research was a great way to innovate and make a name for myself as I set out on my independent career.

*Do you have to teach a lot?* Yes, I teach quite a bit. I teach undergraduate- and graduate-level classes that are part of the traditional chemical engineering core curriculum. I also teach an undergraduate course as part of the microbiology and molecular genetics curriculum. Some might see this as a negative, but I have found quite a few advantages to my teaching role in both departments.

The rest of this article delves into the nuances of finding the right home for your virology research lab and the pros and cons of an unconventional home department. Some of these reflections may be useful even if you are not a virologist, or if you find a great home in a more conventional department for your research program. As with all forms of mutualism, context is key.

## FLEXIBILITY ON THE JOB MARKET

The odds of securing that coveted tenure-track faculty position are slim (3–5). Early-career researchers often move across the country or even across the world for this professional dream. Some researchers do not have that flexibility. You name it—two-body problem, family obligations, or reliable access to healthcare—may limit what is deemed a suitable location.

Applying to non-microbiology departments where you have a legitimate connection will help you increase your options if you are geographically constrained. I applied to chemical engineering departments to broaden my faculty search because of a two-body problem. Some chemical engineering departments have a strong bioengineering/biochemical engineering slant, and you can make a bio-focused lab fit in well if you use quantitative approaches or are heavily invested in technology/tool development. While chemical engineering departments were not always an obvious research fit for me, it made practical sense. Chemical engineering departments have a large undergraduate teaching load they must satisfy and may hire as a result. I could teach almost any class they needed me to.

There may be some obvious disciplines outside of microbiology that could make sense based on your specific training background and research area. If you are considering applying to a non-microbiology department, ask yourself:

*Can I pitch my research in a way that is relevant to the faculty of this department?* This is critical because there are many competitive candidates vying for a single faculty position. “Fit,” while fuzzy, is paramount. You must convince the faculty that you will not only bring in research funding for yourself but also for *them*. They want to know how you will be synergistic to their department and help accelerate *their* research programs through collaboration. This requires more tailoring for a virologist looking to join a non-microbiology department. For me, this meant considering how to make my research proposal more applied—how could we use viruses to inform biotechnology development?

*Can I fulfill the non-research requirements of the position?* You need to convince the department that you fulfill the basic job requirements and that you will not be a burden when it comes to non-research activities. This could be teaching undergraduate or medical school classes, or having a large service role. For me, this was teaching chemical engineering classes. I was confident I could fulfill the teaching needs of most chemical engineering departments.

*Is this department a good fit for you?* Are they open-minded? Are they looking to grow in new directions? Will you be able to recruit students with the right interests and background for your research program? Will they accept that you publish in journals different from them? Will they accept that you attend conferences different from them? Not all of these are knowable upon interviewing or accepting an offer. They involve some risk. But it may be a risk worth taking depending on your particular situation.

Broadening your faculty search also comes with drawbacks. It requires time to tailor your application to some of these departments. Sometimes it can feel like you are really pushing the limits of how much you can stretch your research in a particular direction. If it is not done well, it could come across as naive. I had some very awkward interviews as a result. It can also pose challenges when negotiating a startup package or space. An unconventional department may not be able to offer the startup funds that a microbiology department can offer, or it may not have appropriate space for research that requires biosafety containment. The startup package may be a non-starter, but the space issue can be negotiated within the institution (as was mine). Despite these drawbacks, the advantages of broadening your job search can make it a worthwhile strategy for you.

## RESEARCH

The right home department for you often comes down to research “fit.” As an early-career researcher, it is critical to have colleagues who can critique your grant proposals, mentor you, and collaborate. This is obviously a lot easier to do if there is a good research overlap. In that sense, joining a non-microbiology department could be a huge disadvantage. However, you can fill this gap by reaching out to colleagues at your institution with similar research programs in different departments. If you are considering applying to a non-microbiology department, ask yourself:

*Can I see myself collaborating with any of these people?* This also goes back to the earlier question of whether you can convince *them* that you fit. It is a two-way street.

*Can they provide feedback on my grant proposals?* Do they have any experience applying to the same funding agencies? Even if they do not, they can provide general grantsmanship advice and help you identify funding opportunities that you may not have considered. The default funding agency for many virologists is the NIH, but my chemical engineering colleagues showed me that NSF, DOD, and the DOE/National Lab system also fund fundamental and applied virology research.

Ultimately, the biggest impact on research will be through novel work at the intersection of two disciplines in ways you may not anticipate. During my training, I slowly moved further and further away from traditional chemical engineering research. Returning to the discipline after several years helped me appreciate how virology may benefit from some of the simplest approaches in chemical engineering. While the approach may be simple for chemical engineering, applying it to a complex system like virus infection or cellular dynamics can push a field forward.

This type of interdisciplinary research can pay off in the long term. A key measure of success for early-career researchers is a clear demonstration of independence. This is not just a question of whether you publish papers independent of your former mentors. Are you doing anything truly unique, or are you just “famous mentor lite”? Working at the intersection of virology and chemical engineering has helped me set myself apart from my PhD and postdoc mentors to demonstrate my independence *and* uniqueness. It has also helped me stand out in a crowded field of many talented early-career virologists. Returning to an old discipline or striking out into an adjacent one could help you stand out.

Joining a non-microbiology department can also have some drawbacks. If the student pool you draw from is untrained in virology, they will need more time to catch up. This could prolong a PhD compared to other members of their cohort who join labs more squarely aligned with the core discipline. I deal with this by being honest with students about expectations before they join my lab. The advantage is that a chemical engineering student who wants to study viruses, while rare, is naturally curious and may be willing to take risks. You essentially self-select for trainees who will be your partners in pushing the two disciplines together. Depending on the institution, you could also draw students from different graduate programs to build an interdisciplinary dream team. I took advantage of this at UC Davis and have a mix of biologists and chemical engineers in my group. Depending on the context, an unconventional home department might *enhance* your research program.

## TEACHING

When considering an independent career, a teaching-heavy appointment may seem like a burden or distraction. That valuable time you could be using to develop your research program is instead taken up by prepping courses, office hours, and grading. Medical school appointments might look attractive since the teaching expectation is lower. It is absolutely true that teaching means you have less time to do research, write grants, and write papers. These three elements are a big part of the job description, so how can this even work?

The first thing you need to consider is that you are trading one type of stress for another. A position at a medical school is usually a “soft money” position that involves recovering 50% or more of your salary from grant effort. Your salary will quickly eat into that modular R01 budget. As a result, you are not just writing grants to support your researchers, you are writing grants to support *yourself*. This is stressful in an unpredictable way that depends on the whims of reviewers and federal budgets (or the lack thereof). A position that is teaching heavy is usually a “hard money” position that comes with ~9 months (or more) salary from teaching in the academic year. The salary support you get from grant effort is considered “summer salary.” So, you do spend a lot of time teaching and that gets in the way of writing grants, but you are not necessarily “writing for your life” in the same way. It is a much more predictable type of stress.

Predictability is not the only advantage of a teaching-heavy position. Teaching improves your public speaking. I speak in front of ~100–200 students three to four times a week on topics I never researched myself. Have you ever had to get up in front of ~200 20-somethings and admit you do not know the answer? It is much scarier than any conference or seminar Q&A, where you are literally talking about your work and not the content of an entire discipline.

The key to making teaching-heavy appointments work for you is recognizing that teaching pays long-term research dividends even when it is outside your research area. Some of my most innovative research directions, and what sets me apart from my past advisors and other researchers, is bringing concepts from the classroom into my research. Each class I have taught has enhanced my research program in unexpected ways. If we were to distill the academy to its purest form, integrating teaching with research to advance human knowledge is really the whole point. Teaching helps push the boundaries of what is known because you are constantly pushing that boundary with students in the classroom and the lab. The students in turn push you to look at old concepts in a new way and see connections that you did not realize existed before. If you find yourself in a teaching-heavy position, do not treat teaching as a box to check. Instead, use it as a mechanism to enhance your own research program as I have. How you do this will depend on your specifics, but here are a few examples from my experience.

### Graduate statistical thermodynamics for chemical engineers

This class had absolutely *nothing* to do with my research program when I started my lab. Add to that my only qualification for teaching this class was that I had taken it before and you would be correct to wonder why I would ever teach this class. But I *chose* to teach this class because it put me in front of first year graduate students on day one. I could identify talented and quantitatively minded risk-takers and recruit them to my lab. In the long-term, this class has paid off not just because of student recruitment but also because we now use techniques from statistical thermodynamics to simulate virus-host protein interactions and understand them with atomistic resolution (6, 7). This class has pushed me to grow in ways I could not have anticipated.

### Undergraduate chemical kinetics and reaction engineering

This is a core chemical engineering class for seniors. It is straightforward to teach but completely unrelated to my research program. It is focused on fundamental chemical kinetic concepts that all graduating seniors are expected to know and have not changed for decades. Applying these concepts to classic chemical systems is not innovative in the least. Nonetheless, this class positively influenced our work measuring cellular dynamics on short timescales (8). By applying these classic concepts to a complex biological system, we can open up entirely new fields of research (9).

### Introductory virology

This course is the most closely related to my research area. The breadth of viruses covered in this course has expanded the range of viruses I am knowledgeable about. I feel like a card-carrying virologist now, not just a flavivirus expert. Overcoming my imposter syndrome has been beneficial for my standing in the field, like serving on virology-focused grant review panels. I have also used lectures I prepared for this class to submit grant proposals on new viruses. Sometimes new collaborations require me to quickly become an expert on a new virus family. What better way to become an expert on that virus family than to teach students about it?

The drawbacks of a teaching-heavy appointment stem from the time commitment it draws. It makes you less adaptable in responding to late-breaking funding calls with quick turnaround times. The time commitment drawback is amplified if you are teaching outside your area or returning to a field after a long time away. Preparing to teach chemical engineering classes was time-consuming, even if the material had not changed in decades. The first few offerings of my courses were admittedly not something I was proud of. However, I knew I had the support of incredibly patient colleagues who shared course materials and taught me how to teach these subjects.

The examples I provide are obviously specific to my situation. You should consider how teaching might enhance your research program, even if the teaching is outside of your research area. Do not lose sight that this is why the academy exists.

## CONCLUSIONS

The right home for your independent research group will not only allow you to thrive as you see your research program but also push you to grow in new and unexpected ways. As is the case with virus-host mutualism, the ideal mutualistic professional relationship will be context dependent. By encouraging early-career researchers to take advantage of professional mutualism, I also raise three important follow-up questions for the community as a whole—what are the right conditions for professional mutualism in the academy, how can early-career researchers effectively identify these departments, and how can not-so-early career researchers foster them? While the answers to these questions are not obvious or explicitly articulated here, keep in mind the right home may surprise you. You can do excellent virology research in many different places.
